# Editorial: Insights in: theoretical and philosophical psychology

**DOI:** 10.3389/fpsyg.2023.1268864

**Published:** 2023-09-26

**Authors:** Chiara Fini, Luca Tummolini, Guy Dove, Anna M. Borghi

**Affiliations:** ^1^Department of Dynamic and Clinical Psychology, and Health Studies, Sapienza University of Rome, Rome, Italy; ^2^Institute of Cognitive Sciences and Technologies, Italian National Research Council, Rome, Italy; ^3^Department of Philosophy, University of Louisville, Louisville, KY, United States

**Keywords:** embodied cognition, methodological and theoretical approaches, decision making, health, computational modeling

Multifaceted reflections across different domains of knowledge—ranging from the philosophy of science and new interdisciplinary theoretical backgrounds in the field of psychology and computational neuroscience, to social perception, health and decision-making processes, are objects of the current Theme Issue.

The Theme Issue revolves around three nodes which are brought to the attention of scientists and philosophers as timely issues to deal with:

(i) the necessity of rethinking theoretical and methodological practices in social and life sciences in conformity with the natural evolution of the domains of knowledge,
(ii) the enlargement of (social) embodied perception field by including also psychopathological conditions,
(iii) the definition of the theoretical and ethical borders about the concept of human health and the associated decision-making processes.

The first thematic node is related to the relevance acquired within the scientific community of Open Science practices (e.g., preregistration). In this regard, Jacobucci questions the use of confirmatory and exploratory labels in the era of big data. The author argues that, after the replication crisis, in psychology, confirmatory research is becoming more frequent and increasingly requested. At the same time, the advent of big data leads to the frequent incorporation of exploratory elements. The author argues that applying the simple labels “confirmatory” and “exploratory” can present several limitations and that only the simplest studies can be considered as really confirmatory. Describing their research as confirmatory, researchers tend to hide uncertainty in their theoretical foundations. Overall, according to the author, using the label confirmatory and exploratory has many drawbacks. Instead, the author argues for avoiding the use of the rigid labels of confirmatory and exploratory because they are out of date in a time in which Hypothetic-Deductive research is becoming less frequent. Instead, he advocates for the necessity to explain in a detailed but more flexible way “how replication/generalizability was addressed statistically, the form of reasoning used in developing the study procedures, whether explanation, prediction, or description is the primary aim, and finally, what stage of theory generation, development or appraisal the research line is in.”

Flanked by such aspects, another critical issue is the necessity to build solid theoretical background to be dis(confirmed), allowing consistent advances in knowledge. The reproducibility crisis which has plagued the behavioral sciences in the last years has prompted the development of new scientific practices to avoid repeating the same mistakes of the past. However, in addition to the adoption of the many transparency measures which are intended to improve the quality of data, Witte et al. argue for the importance of developing complementary methods to improve our theory construction. To this end, they propose and assess a new method to evaluate the similarity between a theoretically predicted effect and observations which improve the identification of the underlying theoretical construct. Scientific progress needs to rely on these complementary approaches.

A different approach is explored in Teo's article, which adopts the lens of “white epistemology,” a core tenet of Critical Race Theory—CRT—to argue that psychological science turns out to draw on a race-biased research practice. The article is articulated around a main argument: the impossibility of relying only on the goodness of the scientific method to declare that a research practice has brought clear, reliable, and interpretable outcomes. When treating humanities and psychological issues, epistemological contexts and temporality—which offer the necessary background to interpret psychological differences among social groups—can't be ignored. Results obtained by applying a rigorous scientific method are meaningful only within a sociocultural scaffolding; otherwise, the same results might be erroneously interpreted as underlying “objective” or even worse “eugenetical” differences among populations. As has happened in the past, research outcomes in sociocultural domains—not correctly contextually framed—have offered a scientific justification for social stigma toward some social groups. In conclusion, the de-contextualized interpretation of outcomes obtained with the scientific method, without an appropriate epistemic complexity is not appropriate when studying humans and races.

Regarding the scientific content, instead of the practice, contemporary psychological/neuroscientific knowledge is evolving toward increased cross-field contaminations, with a rapid growth of intertwined hybrid disciplines.

Inspired by the confluence of many diverse approaches into the coherent subdiscipline of robophilosophy, Krageloh et al. make a persuasive case for pushing a similar development in psychological science as well. Given the breakthroughs in AI and robotics and their impact in so many diverse domains, they propose that it is time to give rise to the new field of “robopsychology.” A robopsychology may help organize ongoing streams of research that explore both the impact of these technological innovations on human minds as well the way in which these artifacts may acquire a mind of their own. The rise of a “psychology *of*, *for*, and *by* robots, robotics, and artificial intelligence” is surely a topic that needs to be widely discussed by our community. Regarding the evolution of theoretical approaches in neuroscience, de Wit and Matheson posit as a sensitive topic the re-conceptualization of the best modality of functional mapping. While a weak contextualism, allows to stay at a very abstract level of explanation about a brain area's function, a strong contextualism is open to re-classifications and embraces the context-dependent frame to understand, test and map all the neuro-cognitive mechanisms. Context-dependent neural tuning, neural reuse, degeneracy, plasticity, functional recovery, and the neural correlates of enculturated skills each show that there is a lack of stable mappings between organismal, computational, and neural levels of analysis. Following the authors' perspective, each attempt of mapping discrete neuro-cognitive mechanisms, at neural, computational and phenomenological level, is not feasible. Indeed, recent research shows that behavioral goals and contextual variables affect neural recruitment. A re-conceptualization in cognitive neuroscience about the best modality of functional mapping, appears as necessary. Finally, Ahmad et al. identify promising strands of the Social Exchange Theory—SET—, an inspiring approach to frame social behavior for multidisciplinary domains like social psychology, sociology, anthropology and management science. They assessed the state of the art in the field and developed a systematic approach to identify the most promising directions for future research in SET, which, according to the authors, should move beyond the role of positive reciprocity exchanges. Hopefully, also thanks to their proposal, this long enduring framework will be able to inspire further studies also in the next future.

The second thematic node refers to the theoretical evolution of the (social) embodied perception field. Kim and Effken present a conceptual analysis in which they connect the disturbance of the ecological self and impairments in the perception of affordances. They illustrate the notion of affordance as introduced by Gibson, and argue that when in the presence of affordances, accomplishing successfully intended actions is a sign of autonomy and control in individuals. Without the capability to perceive and actively respond to affordances, the environment stops being meaningful. The authors propose an indirect way to test and validate the notion of affordances, i.e. referring to individuals with mental disorders, and specifically with disorders derived from disturbance of the minimal self (e.g., schizophrenia, post-traumatic stress disorder, and Alzheimer's disease). They characterize this minimal self as “ecological self,” the first form of self we experience in infancy. Following Gibson, they propose that if the perception of self is disturbed, then the ability to attune to exteroceptive information in the environment will be disturbed too. They conclude that impairment in affordance perception might be associated with a disturbance of the self.

Within the psychopathology cluster, eating disorders are particularly prone to be investigated through the lens of (social) embodied perception, as Tramacere suggests in her proposal. Starting from the assumption that (i) when looking at the face of another, the same mirroring circuits–MNS– involved when looking at our own face, are activated, and that (ii) our perception of their face is affected by our feelings toward them, the author contends that it is likely that feelings toward ourselves affect our responses to the mirror image. Thus, our body image would be shaped and represented as a function of our own feelings toward ourselves. In relation with the spontaneous sensorimotor resonance triggered by the other's observation, taking up from the Stern's (2010) notion of the vitality of forms—which capture the expressive style of our actions—Liu et al. propose that this theoretical notion helps explain how we are able to perceive the intentions behind the actions of others. More broadly, the vitality of forms serves as a background condition for our understanding of their mental states.

Then, the third node focuses on health and decision-making processes. Firstly, Binder seeks to reconceptualize how existential suffering is viewed in Western culture. He proposes that it would be better to adopt a concept of “existential health” and thus, to abandon the medical model of pathological suffering. Directly related to the theme of health, Berens and Kim deal with the topic of the controversial debate about the nature of decision-making processes in clinical practice. Specifically, the authors, through a review, list arguments supporting or not the theoretical perspective of risk-assessment decision-making—the idea that the higher the risk involved in a decision, the greater the decisional abilities required for DMC—RS-DMC. In conclusion, most positive defenses of RS-DMC rely on its intuitive appeal, while most criticisms are driven by concern about paternalism or the asymmetry between consent and refusal. Much research about the topic is needed. Finally, a new mathematical model in the Markov process is proposed to explain decision-making dynamics by Bizzarri et al.. The novelty of the proposal relies on the integration of concepts like: tacit knowledge—Pascal's “esprit de finesse”—intuition, emotions, awareness and self- awareness to explain the decision-making processes. Crucially, the obsolete dichotomy between analytical and intuitive (holistic) reasoning is definitely overcome in these mathematical formulations, where both emotional and more implicit factors contribute to decision-making processes. Through the model simulations, it is found that awareness emerges as a dynamic process allowing the decision-maker to switch from habitual to optimal behavior, resulting from a feedback mechanism of self-observation. Furthermore, emotions are embedded in the model as inner factors, possibly fostering the onset of awareness. Importantly, the impact of emotions is re-thought with an explicit dependence on the level of awareness of the individual, so that, the conception that emotion is a noise to be filtered is mitigated by the consideration that it is true at a low state of awareness, and can thus be enhancing for aware individuals. In keeping with this, from a completely different perspective, Kam declines in psychoanalytic terms the following principle: through the “ego inflation” people can take the decision to rationally avoid potential detrimental knowledge and thus to preserve mental wellbeing.

In conclusion, the Theme Issue develops across different dimensions, with the goal to inspire thoughts, ideas, and reflections about methodological and theoretical renewal and progress in the research.

## Author contributions

CF: Writing—original draft. LT: Writing—original draft. GD: Writing—original draft. AB: Writing—original draft.
